# Cyanoacetohydrazide as a Novel Derivatization Agent for the Determination of UHPLC-HRMS Steroids in Urine

**DOI:** 10.3390/molecules29112433

**Published:** 2024-05-22

**Authors:** Azamat Temerdashev, Maria Zorina, Yu-Qi Feng, Elina Gashimova, Victor V. Dotsenko, Vitalij Ioutsi, Sanka N. Atapattu

**Affiliations:** 1 Analytical Chemistry Department, Kuban State University, Krasnodar 350040, Russia; 2 School of Bioengineering and Health, Wuhan Textile University, Wuhan 430415, China; 3 Endocrinology Research Centre, Moscow 117292, Russia; 4 CanAm Bioresearch Inc., Winnipeg, MB R3T 6C6, Canada

**Keywords:** derivatization, non-targeted screening, steroids, LC-HRMS, testosterone, steroidomics, cyanoacetohydrazide

## Abstract

The possibility of cyanoacetohydrazide usage as a novel derivatizing agent is demonstrated in the presented article, and a comparison with hydroxylamine as the most commonly used reagent is provided. Optimal conditions for steroid derivatization with cyanoacetohydrazide are provided. According to the collected data, the maximum yield of derivatives was observed at pH 2.8 within 70 min at 40 °C with 5 ng/mL limit of detection for all investigated analytes. It was shown that cyanoacetohydrazide derivatives produces both syn- and anti-forms as well as hydroxylamine, and their ratios were evaluated and shown in presented work. An efficiency enchantment from two to up to five times was achieved with a novel derivatization reagent. Its applicability for qualitative analysis of steroids in urine was presented at real samples. Additionally, the reproducible fragmentation of the derivatizing agent in collision-induced dissociation offers opportunities for simplified non-targeted steroidomic screening. Furthermore, cyanoacetohydrazide increases ionization efficiency in positive mode, which can eliminate the need for redundant high-resolution instrument runs required for both positive and negative mode analyses.

## 1. Introduction

Steroid hormones play a pivotal role in the regulatory functions of the human body, particularly in processes such as inflammation, cellular metabolism, stress management, and immune function [1]. These hormones are primarily synthesized within endocrine glands, such as gonads and adrenal glands, as well as in the liver or placenta [2]. The elimination of steroid hormones occurs through urinary excretion facilitated by uridine diphosphoglucuronosyl transferases that promote glucuronidation for subsequent elimination [3,4,5,6,7]. The qualitative and quantitative analysis of steroid hormones can be conducted in both conjugated and free forms following hydrolysis. Previously published studies have demonstrated the diagnostic significance of determining conjugated forms, which can serve as distinct indicators for the presence of anabolic steroids or play a crucial role in diagnosing various diseases [8,9,10,11,12]. However, this approach has certain limitations—analyzing conjugated forms necessitates employing liquid chromatography methods combined with mass spectrometry, while deconjugated forms can also be analyzed using the GC-MS(/MS) method [13,14,15]. Currently, the most commonly employed approach for deconjugation involves the utilization of *β*-glucuronidase derived from *E. coli* or *H. pomatia*. This enzymatic hydrolysis method is considered gentle and effectively prevents degradation of analytes in the presence of alkaline or acidic conditions [13,14,15,16,17,18].

Regardless of the form of steroids determined during the analysis, the quantification of steroid hormones at trace levels necessitates preliminary sample concentration. The most commonly employed techniques for this purpose include liquid-liquid extraction [19,20,21], solid-phase extraction [22,23,24], and dispersive liquid-liquid microextraction [25,26,27]. In addition, it is imperative to consider that performing non-targeted steroid screening, encompassing estrogens, androgens, progestins, and corticosteroids simultaneously, poses significant challenges due to the switching the polarity required during analysis. For QTOF instruments specifically, rapid switching introduces notable inaccuracies in determining exact masses [28,29,30,31]. Derivatization can offer a potential solution to address this issue. For instance, the utilization of hydroxylamine enables the generation of oximes for steroid hormones, exhibiting enhanced ionization efficiency in positive ion detection. However, it should be noted that these resulting derivatives have the propensity to form *syn*- and *anti*-forms during HPLC-MS analysis, leading to peak splitting within chromatographic profiles [32,33,34]. Consequently, data processing becomes considerably more challenging when analyzing real samples due to the presence of isobaric components and epiforms with similar retention parameters [33,34]. The main issue of hydroxylamine usage is possibility of producing complex metabolites resulting from Beckmann rearrangement reaction in acidified solutions, which makes hydrazides much preferable candidates for usage as derivatization reagents in LC-MS.

Previously, the advantages and issues of hydrazines usage for derivatization were already described on the 2-hydrazino-1-methylpyridine (HMP) and 2-hydrazino-4-(trifluoromethyl)-pyrimidine (HTP) example in [35]. The authors reported an excellent sensitivity, but they also reported the same issue with *E*- and *Z*-isoforms presence for androgens. It should be noticed that for progesterone-like compounds, the number of possible peaks could be significantly higher because of two groups that could be modified by the derivatization reagent. However, as a result, they resolved the problem with the appearance of a few peaks for one compound.

The aim of this study was to explore the potential use of cyanoacetohydrazide as a derivatization reagent for the analysis of steroids and to compare its effectiveness with hydroxylamine, a conventional reagent widely used for this purpose. Subsequently, we investigated the influence of the amount of derivatizing reagent and pH level on the reaction progress.

## 2. Results

### 2.1. Sample Preparation Optimization

The optimization of derivatization conditions involves the following parameters: pH value, reagent stability, quantity of reagent employed, reaction temperature, and duration.

The first step of the investigation involved establishing a suitable derivatization reagent form for the reaction. This reagent garnered significant interest due to its exceptional solubility in methanol, rendering it a convenient choice for steroid hormone derivatization.

**Figure 1:**
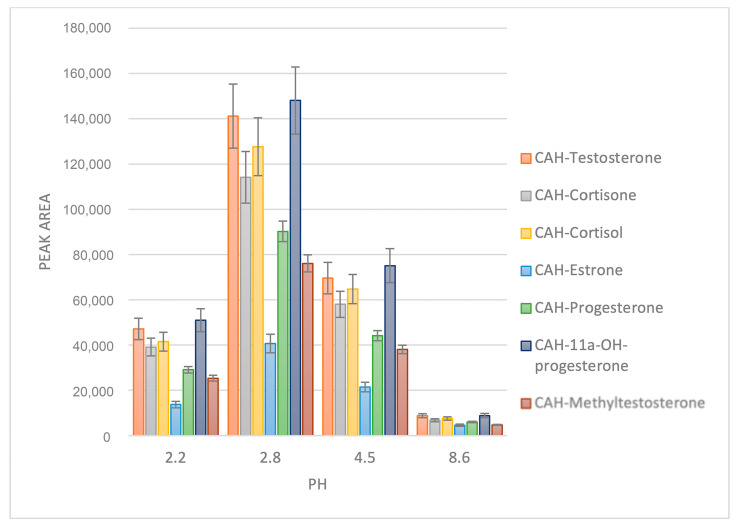
Optimization of the pH for derivatization with cyanoacetohydrazide.

The optimization of derivatization conditions was conducted using a concentration of 100 ng/mL for steroid hormones, which represents an intentionally excessive number of compounds from various classes found in the human body. Consequently, a quantitative yield of derivatives was achieved. As depicted in Figure 1, the optimal pH value for derivatization is determined to be 2.8, corresponding to a formic acid concentration of 0.1% in water. This characteristic renders this reagent highly suitable for LC-MS/MS analysis, as it eliminates the need for additional pH adjustment prior to sample analysis.

**Figure 2:**
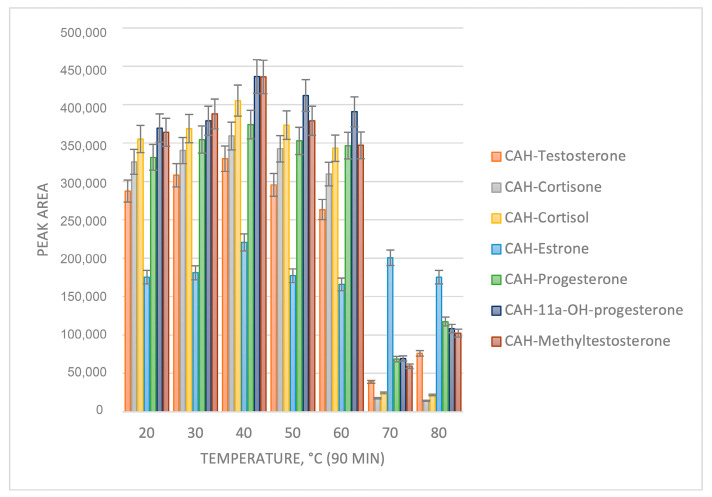
Optimization of the derivatization temperature.

**Figure 3:**
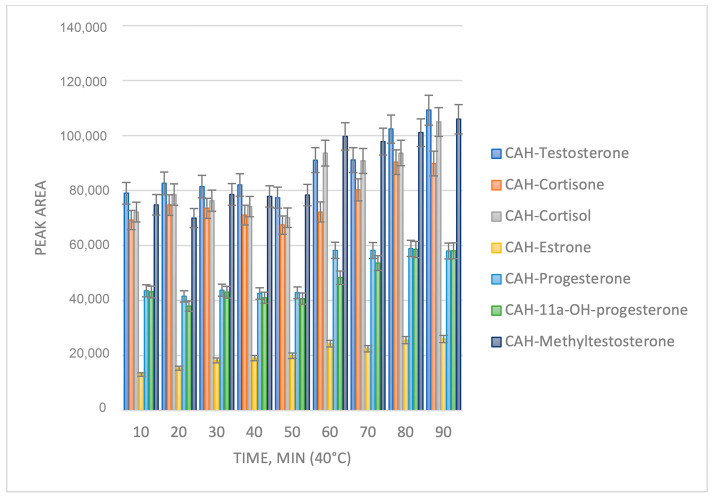
Optimization of the reaction time.

The temperature and reaction time were further investigated (Figure 2 and Figure 3). It can be observed from Figure 2 and Figure 3 that the optimum reaction yield is achieved at a temperature of 40 °C and a reaction time of 70 min.

### 2.2. Chromatographic Separation and MS Detection

The utilization of any novel derivatization agent necessitates an investigation into the resulting chromatographic parameters, including retention on column, separation efficiency, and peak shape, as well as its impact on the analytical signal in terms of ionization efficiency and informative fragmentation in MS/MS mode. 

**Figure 4:**
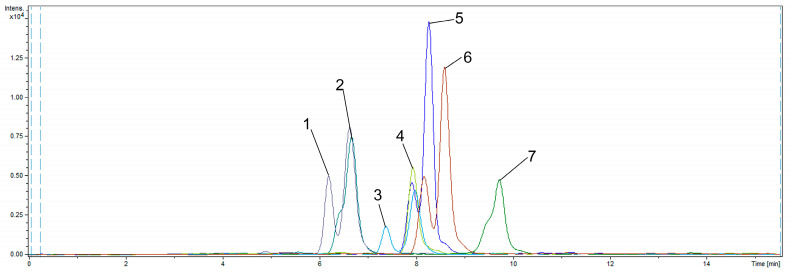
Extracted ion chromatogram of CAH derivatives of steroidal hormones mixture at 10 ng/mL (1—CAH-cortisol; 2—CAH-cortisone; 3—CAH-11α-hydroxyprogesterone; 4—CAH-estrone; 5—CAH-testosterone; 6—CAH-methyltestosterone; 7—CAH-progesterone).

As depicted in Table 1 and Figure 4, cyanoacetohydrazide derivatives exhibit similar analyte retention times; however, the potential formation of syn- and anti-forms during the reaction necessitates their separation.

As can be seen from Figure 4, some syn- and anti-forms of CAH derivatives were not fully separated, and their peaks are too wide for typically observed in UHPLC. To avoid such a picture, additional optimization of separation conditions is required. Proper further separation method optimization could help significantly increase the sensitivity of the analysis because of the better peak shape and their less width than presented in Figure 4.

Similar to oximes of steroidal hormones, both syn- and anti-forms are produced. Although their distribution can be calculated due to similar ionization efficiency (Table 2), determining which exact form corresponds to each peak would require preparative isolation of each component, which necessitates a significant number of components for identification. 

## 3. Discussion

As shown in Table 1 and Table 2, CAH derivatives of the analytes provide two peaks of different forms. In this case control of cross-contamination and stability of chromatographic and mass spectrometric properties become significant. The following criteria were used to prevent false results: retention time shift of the standards and analytes in the real sample should not exceed 0.1 min and theoretical and observed masses should not show difference higher than 5 ppm in full MS scan mode and 100 ppm for MS/MS mode.

To prevent carryover, the autosampler needle was washed before and after sample injection using a water–methanol solution. It was evaluated by analysis of a blank synthetic urine solution after quality control solution (QC) with concentration of analytes at 100 ng/mL. No peaks of target analytes were present in the blank solution analyzed after QC, which indicated a lack of carryover.

Among the key factors determining the feasibility of employing a derivatizing reagent in laboratory practice are enhanced sensitivity of determination, ease of implementation, and associated interfering influences. In the case of cyanoacetohydrazide, its ionization efficiency surpasses that of the native compound but falls short compared to oximes. The comparison between peak areas, indicative of ionization efficiency, is presented in Table 3.

Among the advantages of the novel derivatizing reagent, it is noteworthy that there is a reduction in the presence of multiple peaks corresponding to various combinations of syn- and anti-forms, which can be observed with oximes. However, it should be acknowledged that the existence of several peaks representing a single compound in the chromatogram poses an evident drawback. This issue significantly complicates its application for numerous routine tasks, such as assessing the testosterone:epitestosterone ratio, which holds diagnostic importance in doping control.

**Figure 5:**
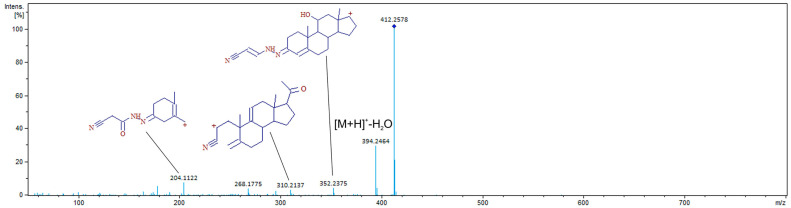
MS/MS spectra of CAH derivative of 11α-hydroxyprogesterone obtained at 15 eV collision energy.

Another important part of usage of novel derivatization reagents is the appearance of repeatable fragments of derivatives, the presence of which could be used as a protentional marker of analytes presence. As can be seen from Figure 5, a fragment with *m/z* 204.1122 could be used as a protentional marker, since its formation is associated with the formation of CAH derivatives. It is interesting that its formation requires the application of a high collision energy.

Simultaneously, the reagent exhibits complete compatibility with HPLC-MS/MS, which can be particularly crucial in certain cases where specific reagents are recommended exclusively for HPLC-MS/MS analysis. Additionally, the stability of the derivatizing reagent was evaluated during the investigation, and consistent results were obtained over a span of one week. Subsequently, a decline in derivative intensities was observed, suggesting potential degradation of the derivatizing reagent. A comparison between derivatization-based and non-derivatization-based approaches also yielded satisfactory outcomes (Table 4). However, a slight overestimation in testosterone concentrations was noticed during derivative analyses due to an unresolved peak present in one form of epitestosterone.

Another crucial aspect of investigating novel derivatizing agents is to compare their sensitivity with that of previously reported effective reagents. In the case of cyanoacetohydrazide, a comparison with hydroxylamine appears most appropriate (Table 5). An optimized condition of derivatization with hydroxylamine was previously reported in [22]. A lower limit of quantification for oximes and CAH derivatives corresponds to the lowest point of the linear range and presented at Table 5.

As can be seen in Table 5, the limit of quantification for analytes was established at 5 ng/mL, which is closer in range to oximes.

## 4. Materials and Methods

### 4.1. Chemicals

Standards of testosterone, methyltestosterone (St. Lois, MO, USA) (internal standard, **IS**), progesterone, cortisone, cortisol, estrone, and 11α-OH-progesterone (>99%) were purchased from Sigma-Aldrich (St. Lois, MO, USA); PLC-grade acetonitrile (“Biosolve”, Jerusalem, Israel), 18.2 MΩwater (Milli-Q, Millipore, Molsheim, France), and formic acid (98%, Acros Organics, Geel, Belgium) were used as the mobile phase. Methanol of HPLC grade was purchased from Vecton (Saint-Petersburg, Russia). Potassium carbonate (≥99%, Vecton, Saint-Petersburg, Russia), potassium bicarbonate (≥99%, Vecton, Saint-Petersburg, Russia), sodium hydroxide (≥99%, Reactive, Saint-Petersburg, Russia), sodium tetraborate (≥99%, Vecton, Saint-Petersburg, Russia), sodium hydrogen phosphate (≥99%, Vecton, Saint-Petersburg, Russia), potassium dihydrogen phosphate (≥99%, Vecton, Saint-Petersburg, Russia), ammonium acetate (≥99%, Vecton, Saint-Petersburg, Russia), and acetic acid were used for the preparation of buffer solutions with pH 10.5, 9.5, 6.5, and 4.5, respectively. 

### 4.2. Instrumentation

A Bruker MaXis Impact (Bruker Daltonik GmbH, Bremen, Germany) quadrupole-time-of-flight mass spectrometer (Q-TOF) high-resolution mass spectrometer (**HRMS**) equipped with an electrospray ionization (ESI) source coupled with an ultra-high performance liquid chromatography Bruker Elute system (UHPLC) with a Phenomenex Kinetex C18 (100 mm × 2.1 mm, 1.7 μm) column and an appropriate guard column was used for the chromatographic separation. A two-component system of acetonitrile (A)–0.1% formic acid in water was (B) used as the mobile phase. The gradient elution program was as follows: 0.0–0.5 min 2% A, 98% B, 0.5–14.5 min 100% A, 0% B, 14.5–17.5 min 100% A, 0% B, 17.5–17.6 min 2% A, 98% B, 17.6–23.0 min 2% A, and 98% B.

The injection volume was 10 μL. The flow rate was held constant at 0.3 mL/min, and the column thermostat temperature was 40 °C. The voltage at the ionization source was 3.5 kV, drying gas flow rate was 8 L/min, spray gas pressure was 2 bar, temperature of the ionization source was 250 °C, mass scanning range (*m/z*) was 50–600, and scanning speed was 3 Hz. Data acquisition and analysis were performed with Bruker Compass HyStar 4.1 and Bruker Data Analysis 4.4 software, respectively. A full scan mode was used for data collection and for qualitative analysis to prevent loss of the sensitivity.

### 4.3. Urine Sample Preparation

Urine samples obtained from volunteers (males and females aged between 20 and 45) were used to prepare calibration curves and validate the procedure. The samples were preserved with sodium azide and then stored at −20 °C prior to analysis. 

The following sample preparation was used: 1.75 mL of phosphate buffer (pH 6.5), 250 µL of a methanol solution of methyltestosterone with a concentration of 2 µg/mL, and 30 µL of *E. coli β*-glucuronidase enzyme, which were added to 3 mL of sample, mixed thoroughly, and incubated at 50 °C for 30 min. Then, 3 mL of diethyl ether and 2 g of Na_2_SO_4_ were added, vortexed for 2 min, centrifuged for 5 min at 4000 rpm, and placed in a cryostat until the aqueous layer completely froze. The ether layer was evaporated at 60 °C, and redissolved in 200 µL of methanol and cyanoacetohydrazide solution, containing 0.1% of formic acid with further incubation for 70 min at 40 °C. The samples were analyzed after cooling to room temperature.

### 4.4. Preparation of Standard and Stock Solutions

Stock standard solutions of steroidal hormones with concentration of 1 mg/mL were prepared in methanol. Working solutions of standards were prepared by dilution of stock solutions with 0.1% formic acid in water. Working solutions of the derivatization agent cyanoacetohydrazide was obtained by dissolving appropriate reagent weights in methanol to achieve 50 mg/mL concentration. Quality control (QC) solutions containing steroids at high (100 ng/mL), medium (50 ng/mL), and low (10 ng/mL) concentrations were prepared from working solutions.

## 5. Conclusions

The potential use of cyanoacetohydrazide as a derivatization reagent was discussed, and the optimization of sample preparation conditions was presented. It was demonstrated that the maximum yield of derivatives was observed at pH 2.8 within 70 min at 40 °C. A comparative study between the novel derivatization reagent and hydroxylamine was conducted, highlighting its applicability to real urine samples. The limit of detection for all target compounds was determined to be 2.5 ng/mL, which is significantly higher than that for oximes of steroids. However, the limit of quantification for analytes was established at 5 ng/mL, which is closer in range to oximes. The main issues and advantages associated with cyanoacetohydrazide were thoroughly discussed.

## Figures and Tables

**Table 1:** Comparison of retention parameters of CAH derivatives, HA-derivatives, and native steroidal hormones.

Compound	Retention Time, min	[M + H]^+^ Theoretical, *m/z*	[M + H]^+^ Observed, *m/z*	Mass Error, ppm
CAH-testosterone peak 1	7.9	370.2489	370.2486	0.81
CAH-testosterone peak 2	8.3			
CAH-cortisone peak 1	6.4	442.2336	442.2339	−0.68
CAH-cortisone peak 2	6.7			
CAH-cortisol peak 1	6.2	444.2493	444.2492	0.23
CAH-cortisol peak 2	6.6			
CAH-estrone	7.9	352.2020	352.2016	1.14
CAH-progesterone peak 1	9.5	396.2656	396.2657	−0.25
CAH-progesterone peak 2	9.7			
CAH-11α-hydroxyprogesterone peak 1	7.4	412.2595	412.2599	−0.97
CAH-11α-hydroxyprogesterone peak 2	8.0			
CAH-methyltestosterone peak 1	8.2	384.2656	384.2651	1.30
CAH-methyltestosterone peak 2	8.6			
Testosterone	8.0	289.2162	289.2161	0.35
Cortisone	6.3	361.2010	361.1995	4.15
Cortisol	6.2	363.2166	363.2165	0.27
Progesterone	9.7	315.2319	315.2319	0.00
Estrone	7.9	[M − H]^−^: 269.1547	[M − H]^−^: 269.1540	2.60
11α-hydroxyprogesterone	7.4	331.2268	331.2256	3.62
Methyltestosterone	8.4	303.2319	303.2312	2.31
HA-testosterone peak 1	8.1	304.2271	304.2267	1.21
HA-testosterone peak 2	8.2			
HA-cortisone peak 1	6.3	391.2227	391.2225	0.34
HA-cortisone peak 2	6.4			
HA-cortisol peak 1	6.2	393.238	393.2378	0.55
HA-cortisol peak 2	6.3			
HA-estrone	8.0	286.1802	268.1797	1.75
HA-progesterone peak 1	9.7	345.2537	345.2525	3.38
HA-progesterone peak 2	9.8			
HA-11α-hydroxyprogesterone peak 1	7.6	361.2486	361.2476	2.79
HA11α-hydroxyprogesterone peak 2	7.7			
HA-methyltestosterone peak 1	8.4	318.2428	318.2418	3.02
HA-methyltestosterone peak 2	8.5			

**Table 2:** Syn- and anti-forms of the analyte ratios.

Compounds Ratio	Peaks Area Ratio
CAH-testosterone (peak 2)/CAH-testosterone (peak 1)	3.2
CAH-cortisone (peak 2)/CAH-cortisone (peak 1)	3.6
CAH-cortisol (peak 2)/CAH-cortisol (peak 1)	3.6
CAH-cortisol (peak 2)/CAH-cortisol (peak 1)	2:1
CAH- progesterone (peak 2)/CAH- progesterone (peak 1)	2.4
CAH-11α-hydroxyprogesterone (peak 2)/ CAH-11α-hydroxyprogesterone (peak 1)	2.6
CAH-methyltestosterone (peak 2)/ CAH-methyltestosterone (peak 1)	2

**Table 3:** Comparison of the responses of derivatives against native steroids.

Compounds Ratio	Peaks Area Ratio
CAH-testosterone (peak 2)/Testosterone	5.3
CAH-cortisone (peak 2)/Cortisone	4
CAH-cortisol (peak 2)/Cortisol	3.8
CAH-progesterone (peak 2)/Progesterone	1.4
CAH-11α-hydroxyprogesterone (peak 2)/11α-hydroxyprogesterone	2.4
CAH-methyltestosterone (peak 2)/Methyltestosterone	3

**Table 4:** Comparison of the real sample analysis results.

Compound	Concentration, ng/mL	
	Native	CAH-Derivatives
Testosterone	12 ± 2	16 ± 3
Cortisone	10 ± 2	12 ± 2
Cortisol	4.2 ± 0.8	5.5 ± 1.0

**Table 5:** Comparison of the oximes and CAH derivatives of the steroids.

Compound	Oximes			CAH-Derivatives		
	LOD, ng/mL	Linear Range, ng/mL	R^2^	LOD, ng/mL	Linear Range, ng/mL	R^2^
Testosterone	0.25	1.0–100	0.9987	2.5	5–100	0.9991
Cortisone	0.5	2.5–100	0.9962	2.5	5–100	0.9973
Cortisol	0.5	2.5–100	0.9976	2.5	5–100	0.9974
Progesterone	0.25	1.0–100	0.9945	2.5	5–100	0.9967
Estrone	0.5	2.5–100	0.9912	2.5	5–100	0.9945
11α-hydroxyprogesterone	0.25	1.0–100	0.9889	2.5	5–100	0.9925

## Data Availability

Data is contained within the article.

## References

[1] Son H.H., Yun W.S., Cho S.H. (2020). Development and validation of an LC-MS/MS method for profiling 39 urinary steroids (estrogens, androgens, corticoids, and progestins). Biomed. Chromatogr..

[2] Wang R., Hartmann M.F., Wudy S.A. (2021). Targeted LC–MS/MS analysis of steroid glucuronides in human urine. J. Steroid Biochem. Mol. Biol..

[3] Wells P.G., Mackenzie P.I., Chowdhury J.R., Guillemette C., Gregory P.A., Ishii Y., Hansen A.J., Kessler F.K., Kim P.M., Chowdhury N.R. (2004). Glucuronidation and the UDP-glucuronosyltransferases in health and disease. Drug Metab. Dispos..

[4] Kalogera E., Pistos C., Provatopoulou X., Athanaselis S., Spiliopoulou C., Gounaris A. (2013). Androgen glucuronides analysis by liquid chromatography tandem-mass spectrometry: Could it raise new perspectives in the diagnostic field of hormone-dependent malignancies?. J. Chromatogr. B Anal. Technol. Biomed. Life Sci..

[5] Falk R.T., Xu X., Keefer L., Veenstra T.D., Zieger R.G. (2008). A Liquid Chromatography-Mass Spectrometry Method for the Simultaneous Measurement of Fifteen Urinary Estrogens and Estrogen Metabolites: Assay Reproducibility and Inter-individual Variability. Cancer Epidemiol. Biomark. Prev..

[6] Mueller J.W., Gilligan L.C., Idkowiak J., Arlt W., Foster P.A. (2015). The regulation of steroid action by sulfation and desulfation. Endocr. Rev..

[7] Labrie F., Bélanger A., Bélanger P., Bérubé R., Martel C., Cusan L., Gomez J., Candas B., Castiel I., Chaussade V. (2006). Androgen glucuronides, instead of testosterone, as the new markers of androgenic activity in women. J. Steroid Biochem. Mol. Biol..

[8] Storbeck K.H., Schiffer L., Baranowski E.S., Chortis V., Prete A., Barnard L., Gilligan L.C., Taylor A.E., Idkowiak J., Arlt W. (2019). Steroid Metabolome Analysis in Disorders of Adrenal Steroid Biosynthesis and Metabolism. Endocr. Rev..

[9] Olesti E., Boccard J., Visconti G., González-Ruiz V., Rudaz S. (2021). From a single steroid to the steroidome: Trends and analytical challenges. J. Steroid Biochem. Mol. Biol..

[10] Emond J.P., Lacombe L., Caron P., Turcotte V., Simonyan D., Aprikian A., Saad F., Carmel M., Chevalier S., Guillemette C. (2021). Urinary oestrogensteroidome as an indicator of the risk of localised prostate cancer progression. Br. J. Cancer.

[11] Girel S., Markin P.A., Tobolkina E., Boccard J., Moskaleva N.E., Rudaz S., Appolonova S.A. (2024). Comprehensive plasma steroidomics reveals subtle alterations of systemic steroid profile in patients at different stages of prostate cancer disease. Sci. Rep..

[12] Shackleton C., Pozo O.J., Marcos J. (2018). GC/MS in recent years has defined the normal and clinically disordered steroidome: Will it soon be surpassed by LC/Tandem MS in This Role?. J. Endocr. Soc..

[13] Temerdashev A., Nesterenko P., Dmitrieva E., Zhurkina K., Feng Y.Q. (2022). GC-MS/MS Determination of Steroid Hormones in Urine Using Solid-Phase Derivatization as an Alternative to Conventional Methods. Molecules.

[14] Hansen M., Jacobsen N.W., Nielsen F.K., Björklund E., Styrishave B., Halling-Sørensen B. (2011). Determination of steroid hormones in blood by GC-MS/MS. Anal. Bioanal. Chem..

[15] Weitzel K.-M. (2011). Bond-Dissociation Energies of Cations—Pushing the Limits to Quantum State Resolution. Mass Spectrom. Rev..

[16] Temerdashev A., Dmitrieva E., Podolskiy I. (2021). Analytics for steroid hormone profiling in body fluids. Microchem. J..

[17] Matysik S., Liebisch G. (2017). Quantification of steroid hormones in human serum by liquid chromatography-high resolution tandem mass spectrometry. J. Chromatogr. A.

[18] Liere P., Schumacher M. (2015). Mass spectrometric analysis of steroids: All that glitters is not gold. Expert Rev. Endocrinol. Metab..

[19] Ney L.J., Felmingham K.L., Bruno R., Matthews A., Nichols D.S. (2021). Chloroform-based liquid-liquid extraction and LC–MS/MS quantification of endocannabinoids, cortisol and progesterone in human hair. J. Pharm. Biomed. Anal..

[20] Domenech-Coca C., Mariné-Casadó R., Caimari A., Arola L., del Bas J.M., Bladé C., Rodriguez-Naranjo M.I. (2019). Dual liquid-liquid extraction followed by LC-MS/MS method for the simultaneous quantification of melatonin, cortisol, triiodothyronine, thyroxine and testosterone levels in serum: Applications to a photoperiod study in rats. J. Chromatogr. B Anal. Technol. Biomed. Life Sci..

[21] Keevil B.G. (2013). Novel liquid chromatography tandem mass spectrometry (LC-MS/MS) methods for measuring steroids. Best Pract. Res. Clin. Endocrinol. Metab..

[22] Dmitrieva E.V., Temerdashev A.Z., Zorina M.O., Feng Y.Q., Nesterenko P.N. (2022). Solid-phase analytical derivatization as a tool for the quantification of steroid hormones in human urine with HPLC-Q-ToF detection. J. Pharm. Biomed. Anal..

[23] Dévier M.H., Labadie P., Togola A., Budzinski H. (2010). Simple methodology coupling microwave-assisted extraction to SPE/GC/MS for the analysis of natural steroids in biological tissues: Application to the monitoring of endogenous steroids in marine mussels *Mytilus* sp.. Anal. Chim. Acta.

[24] Márta Z., Bobály B., Fekete J., Magda B., Imre T., Mészáros K.V., Bálint M., Szabó P.T. (2018). Simultaneous determination of thirteen different steroid hormones using micro UHPLC-MS/MS with on-line SPE system. J. Pharm. Biomed. Anal..

[25] El-Deen A.K., Shimizu K. (2019). Deep eutectic solvent as a novel disperser in dispersive liquid-liquid microextraction based on solidification of floating organic droplet (DLLME-SFOD) for preconcentration of steroids in water samples: Assessment of the method deleterious impact on the environment using Analytical Eco-Scale and Green Analytical Procedure Index. Microchem. J..

[26] Dmitrieva E.V., Temerdashev A.Z., Osipova A.K. (2021). Determination of Ketosteroids in Human Urine Using Dispersive Liquid-Liquid Microextraction and Ultra High-Performance Liquid Chromatography-High Resolution Mass Spectrometry. J. Anal. Chem..

[27] Srivastava A., Godbole M.M., Shrivastava A. (2022). Estimation of Steroid Hormones in Biological Samples Using Micro Extraction and Advanced Chromatography Techniques. Austin J. Anal. Pharm. Chem..

[28] Olesti E., Garcia A., Rahban R., Rossier M.F., Boccard J., Nef S., González-Ruiz V., Rudaz S. (2020). Steroid profile analysis by LC-HRMS in human seminal fluid. J. Chromatogr. B Anal. Technol. Biomed. Life Sci..

[29] Elmongy H., Masquelier M., Ericsson M. (2020). Development and validation of a UHPLC-HRMS method for the simultaneous determination of the endogenous anabolic androgenic steroids in human serum. J. Chromatogr. A.

[30] Davis D.E., Leaptrot K.L., Koomen D.C., May J.C., Cavalcanti G.D.A., Padilha M.C., Pereira H.M.G., McLean J.A. (2021). Multidimensional Separations of Intact Phase II Steroid Metabolites Utilizing LC-Ion Mobility-HRMS. Anal. Chem..

[31] Fabresse N., Grassin-Delyle S., Etting I., Alvarez J.C. (2017). Detection and quantification of 12 anabolic steroids and analogs in human whole blood and 20 in hair using LC-HRMS/MS: Application to real cases. Int. J. Legal Med..

[32] Xu B., Jia P., Cai J., Gu L., Yan H., Zhao H., Qin S. (2022). Simultaneous quantitative analysis of seven steroid hormones in human saliva: A novel method based on O-ethylhydroxylamine hydrochloride as derivatization reagent. Rapid Commun. Mass Spectrom..

[33] Song Z., Gao H., Xie W., Sun Q., Liang K., Li Y. (2021). Quantitative MALDI-MS assay of steroid hormones in plasma based on hydroxylamine derivatization. Anal. Biochem..

[34] Liu Q., Chi Q., Fan R.T., Tian H.D., Wang X. (2019). Quantitative-Profiling Method of Serum Steroid Hormones by Hydroxylamine-Derivatization HPLC–MS. Nat. Prod. Bioprospect..

[35] Faqehi A.M., Denham S.G., Naredo G., Cobice D.F., Khan S., Simpson J.P., Sabil G., Upreti R., Gibb F., Homer N.Z. (2021). Derivatization with 2-hydrazino-1-methylpyridine enhances sensitivity of analysis of 5α-dihydrotestosterone in human plasma by liquid chromatography tandem mass spectrometry. J. Chromatogr. A.

